# A Desmethylphosphinothricin Dipeptide Derivative Effectively Inhibits *Escherichia coli* and *Bacillus subtilis* Growth

**DOI:** 10.3390/biom13101451

**Published:** 2023-09-26

**Authors:** Maxim A. Khomutov, Fabio Giovannercole, Laura Onillon, Marija V. Demiankova, Byazilya F. Vasilieva, Arthur I. Salikhov, Sergey N. Kochetkov, Olga V. Efremenkova, Alex R. Khomutov, Daniela De Biase

**Affiliations:** 1 Engelhardt Institute of Molecular Biology, Russian Academy of Sciences, Vavilov Street 32, 119991 Moscow, Russia; makhomutov@mail.ru (M.A.K.); asalihov93@gmail.com (A.I.S.); kochet@eimb.ru (S.N.K.); 2 Department of Medico-Surgical Sciences and Biotechnologies, Sapienza University of Rome, Corso della Repubblica 79, I-04100 Latina, Italy; fabio.giovannercole@gmail.com (F.G.); laura.onillon@ifremer.fr (L.O.); 3 Gause Institute of New Antibiotics, Bol’shaya Pirogovskaya 11, 119021 Moscow, Russia; mary_bunny@mail.ru (M.V.D.); bfvas@yandex.ru (B.F.V.); ovefr@yandex.ru (O.V.E.)

**Keywords:** antibacterial, glutamate metabolism, antimicrobial resistance, central metabolism, dipeptide permeases, phosphorus-containing glutamate analogues

## Abstract

New antibiotics are unquestionably needed to fight the emergence and spread of multidrug-resistant bacteria. To date, antibiotics targeting bacterial central metabolism have been poorly investigated. By determining the minimal inhibitory concentration (MIC) of desmethylphosphinothricin (Glu-γ-P_H_), an analogue of glutamate with a phosphinic moiety replacing the γ-carboxyl group, we previously showed its promising antibacterial activity on *Escherichia coli*. Herein, we synthetized and determined the growth inhibition exerted on *E. coli* by an *L*-Leu dipeptide derivative of Glu-γ-P_H_ (*L*-Leu-*D*,*L*-Glu-γ-P_H_). Furthermore, we compared the growth inhibition obtained with this dipeptide with that exerted by the free amino acid, i.e., Glu-γ-P_H_, and by their phosphonic and non-desmethylated analogues. All the tested compounds were more effective when assayed in a chemically-defined minimal medium. The dipeptide *L*-Leu-*D*,*L*-Glu-γ-P_H_ had a significantly improved antibacterial activity (2 μg/mL), at a concentration between the non-desmethytaled (0.1 μg/mL) and the phosphonic (80 μg/mL) analogues. Also, in *Bacillus subtilis*, the dipeptide *L*-Leu-*D*,*L*-Glu-γ-P_H_ displayed an activity comparable to that of the antibiotic amoxicillin. This work highlights the antibacterial relevance of the phosphinic pharmacophore and proposes new avenues for the development of novel antimicrobial drugs containing the phosphinic moiety.

## 1. Introduction

**Figure 1:**
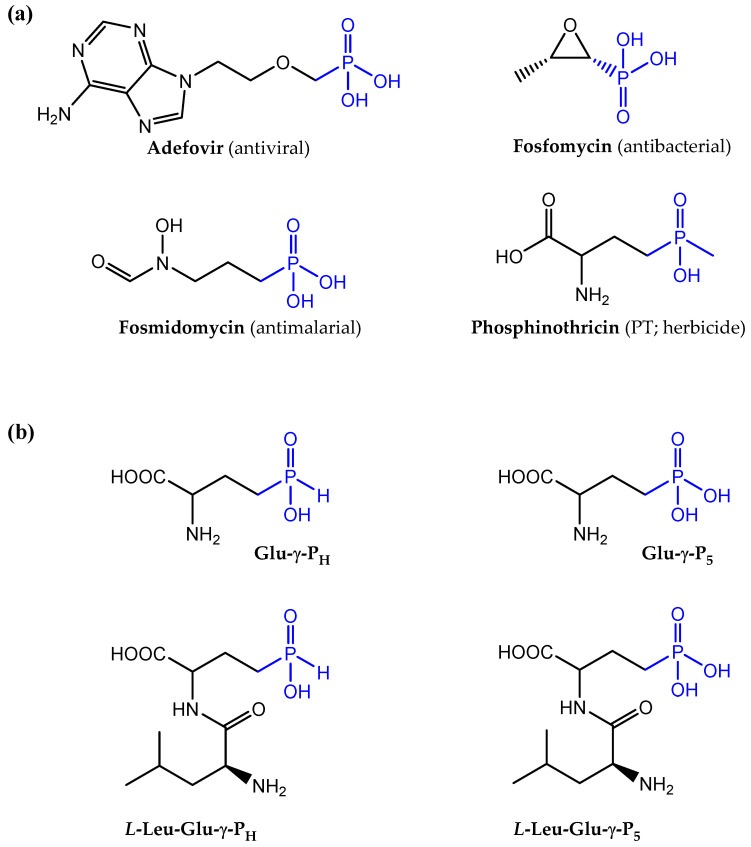
Chemical structures of molecules containing phosphonic and phosphinic groups. (**a**) Notable examples of molecules containing phosphonic and phosphinic groups (in blue) with a pharmacological activity. Only phosphinothricin (PT) belongs to the class of the phosphinic compounds, even more rare in nature. (**b**) Chemical structures of *H*-phosphinic (P_H_) and phosphonic (P_5_) analogues of glutamate included in this study, Glu-γ-P_H_ and Glu-γ-P_5_, respectively, and, below them, the two corresponding leucine-containing dipeptides.

Organic molecules containing phosphonic and phosphinic acidic groups are unusual, though naturally occurring compounds [1,2,3,4,5,6]. They are characterized by the presence of a carbon–phosphorous (C-P) bond, which is rather rare in nature. In particular, phosphonates contain a single C–P bond, whereas phosphinates contain either two such bonds (i.e., C-P-C) or C-P-H bonds. For both types of compounds, the rest of the valences on the phosphorous atom are engaged in bonding oxygen. The C-P bond, unlike the C-O-P bonds, such as those occurring in the more common organic phosphate esters and anhydrides, has unique features, including a remarkable stability against enzymatic (i.e., it cannot be cleaved by hydrolyses) or chemical cleavage, such as acid/base hydrolysis. Notably, the phosphinic and phosphonic moieties structurally mimic phosphate esters, carboxylates and tetrahedral intermediates occurring during carboxyl group transformations [3,4]. This also explains why many of these compounds act as substrates or competitive inhibitors of the targeted enzymes. Important examples of these classes of molecules include compounds of natural origin, such as the antibiotics fosfomycin [6], dehydrophos and plumbemycin, the antimalarial compounds fosmidomycin and FR-900098, the antifungals rhizocticins, and the herbicide phosphinothricin (PT; also known as glufosinate) [3,4], as well as the chemically synthesized antivirals Adefovir and Tenofovir, which are successfully employed to treat hepatitis B infections [7]. Examples of such molecules are shown in Figure 1a.

Given that some of the natural compounds mentioned above may also be toxic to the producing micro-organism, most of the above listed molecules are naturally synthetized as di- or tri-peptide precursors, which can enter the target cell through dipeptide or oligopeptide permeases, namely, Dpp and Opp, respectively [3,4,8,9]. Once internalized, they are cleaved by cytosolic peptidases that cause the release of the active C-P-containing amino acid. This mechanism has been referred as a “Trojan horse” or pro-drug strategy [3,4,8,9]. A remarkable example is phosphinothricin (PT; glufosinate; Figure 1a), a non-proteinogenic amino acid, which was initially isolated as a bioactive component of the tripeptide Bialaphos, phosphinothricyl-*L*-alanyl-*L*-alanine. PT is a phosphinic analogue of *L*-glutamate, with a phosphinic moiety (C-PO_2_HCH_3_) replacing the glutamate γ-carboxyl group. By mimicking the γ-phosphorylated intermediate of glutamate formed during the first step of the reaction, the pyrophosphonate of PT formed during the reaction was demonstrated to act as a competitive inhibitor of the enzyme glutamine synthetase (GS), which catalyzes the ATP-dependent formation of glutamine starting from glutamate and ammonia, [10,11]. In plants, the inhibition of GS leads to a rapid accumulation of intracellular ammonium ion (NH_4_^+^), which perturbs pH homeostasis and leads to cell death. Hence, PT and the PT-containing tripeptides, Bialaphos or Phosalacine (i.e., PT-*L*-alanyl-*L*-leucine-), exhibit strong herbicidal activity [3,5,12,13]. In addition to the well-established herbicidal effect, Bialaphos and a PT-containing dipeptide, *L*-Leu-*L*-PT, were remarkably effective in clinical isolates of *Klebsiella pneumoniae*, which displayed resistance to more than 20 commercial antibiotics belonging to different classes [14].

Less investigated than PT and Bialaphos is the PT desmethylated on the phosphinic moiety (*L*-2-amino-4-(hydroxy)-phosphinylbutyric acid; hereafter referred to as *L*-Glu-γ-P_H_; Figure 1b), which is also an analogue of *L*-glutamate, that carries the more rarely occurring *H*-phosphinic group, i.e., C-P(O)(OH)H. *L*-Glu-γ-P_H_ was originally isolated as an intermediate in the biosynthesis of Bialaphos in *Streptomyces hygroscopicus* [15] and more recently in the free form in *Nonomureae* sp. NRRL B-24552 [1]. In *S. hygroscopicus*, *L*-Glu-γ-P_H_ was shown to accumulate and inhibit growth in the mutant form of this micro-organism where the Bialaphos biosynthetic pathway was blocked [16]. Indeed, to protect themselves from the action of the antibiotics they synthetize, the micro-organisms producing it (i.e., *Streptomyces*) initially inactivate PT, or desmethylphosphinothricin, by acetylation [17], then the acetylated precursors are used for the synthesis of the tripeptide Bialaphos, which is released into the extracellular environment. Acute toxicity studies conducted with Glu-γ-P_H_ demonstrated very low toxicity of the compound. The LD_50_ were 2740 mg/kg following oral administration and 1180 mg/kg following intraperitoneal injection in mice and were more than 5000 mg/kg following oral administration in rats [18]. Notably, seven days post-administration, no histological changes were observed in the surviving animals [18].

To date, the mechanism of antibacterial activity of *L*-Glu-γ-P_H_ remains unknown; however, we demonstrated that the *H*-phosphinic group of *L*-Glu-γ-P_H_ is a bioisostere of carboxylates, and that the desmethylated phosphinic compounds derived from it (i.e., the *H*-phosphinic analogues of GABA and succinate) can be recognized and metabolized just as the substrate by the relevant enzymes [19]. We also found that only the *L*-isomer of Glu-γ-P_H_ displays an antibacterial activity, which implies that the compound is indeed metabolized and leads to the formation of intermediate(s) eventually responsible for the observed antibacterial activity [19]. To the best of our knowledge, peptides containing amino acids with a *H*-phosphinic group in a distal position from the carboxyl group have never been investigated as antibacterials. Here, we studied the inhibition of growth caused by the dipeptide *L*-Leu-*D*,*L*-Glu-γ-P_H_ on both *Escherichia coli* and *Bacillus subtilis*, as representatives of Gram-negative and Gram-positive bacteria, respectively. We compared its activity with that of *D*,*L*-PT and *L*-Leu-*D*,L-PT, its dipeptide derivative, as well as with the corresponding phosphonic analogues of glutamate (i.e., Glu-γ-P_5_ and *L*-Leu-*D*,L-Glu-γ-P_5_; Figure 1b). Our data suggest that the incorporation of Glu-γ-P_H_ in a dipeptide significantly improves the penetration of the molecule, thus enhancing its antibacterial activity and potential use for treating bacterial infections caused by different microorganisms. This work represents additional evidence that phosphinic compounds can be regarded as interesting molecules with antibacterial activity, as recently proposed for the PT-derived dipeptide on multidrug resistant clinical isolates of *K. pneumoniae* [14].

## 2. Materials and Methods

### 2.1. Materials

*D*,L-Glu-γ-P_H_ was synthesized as described in [20]; *N*-(benzyloxycarbonyl)-*L*-leucine *N*-hydroxysuccinimide ester (Z-*L*-Leu-OSu) was prepared according to [21] and was recrystallized from *i*-PrOH before use. *L*-2-Amino-4-phosphonobutyric acid (L-AP4) was obtained from Santa Cruz Biotechnology and was recrystallized from H_2_O-EtOH before use; the Amoxicillin (2.0 μg per disk), was from Becton, Dickinson & Co. (Franklin Lakes, NJ, USA).

Synthesis of *L*-Leucyl-PT is described in detail elsewhere [14].

Agar agar powder No. 1 for the bacteriology was from LobaChemie (Tarapur, India). All other reagents, salts and solvents were of the highest purity and used as supplied by Sigma-Aldrich (Burlington, MA, USA) and Acros (Cedar Rapids, IA, USA).

TLC was carried out on plastic sheet Cellulose F_254_ (Merck, Darmstadt, Germany) in *i*-PrOH–25% NH_4_OH–H_2_O = 7:1:2. *L*-Leu-*D*,*L*-Glu-γ-P_H_ and *L*-Leu-*D*,*L*-Glu-γ-P_5_ were detected on TLC plates following staining with ninhydrin (0.4% in acetone).

Ion-exchange chromatography was carried out on Dowex 50W-X8, H^+^-form, 100–200 mesh (BioRad, Hercules, CA, USA) using water for the elution.

NMR spectra were recorded on a Bruker AM-300 (300.13 MHz for 1H, 75.43 MHz for ^13^C, and 121.44 MHz for ^31^P) using D_2_O as a solvent with sodium 3-trimethyl-1-propanesulfonate (DSS) as the internal, or 85% H_3_PO_4_ as the external standards. Chemical shifts are given in parts per million (ppm), while the letter “*J*” indicates spin–spin coupling constants which are given in Hertz (Hz).

### 2.2. Synthesis of L-Leucyl-D,L-Glu-γ-P_H_

A solution of *N*-Cbz-*L*-Leu-OSu (1.08 g, 3.0 mmol) in 1,2-dimethoxyethane (5 mL) was added to a solution containing *D*,*L*-Glu-γ-P_H_ (500 mg, 3.0 mmol), NaHCO_3_ (127 mg, 1.5 mmol), Na_2_CO_3_ (158 mg, 1.5 mmol) in 1.0 M NaOH (6 mL), water (1 mL) and 1,2-dimethoxyethane (1 mL), and the reaction mixture was stirred overnight at 20 °C. The reaction mixture was concentrated in vacuo, the residue was then dissolved in water (15 mL), acidified with 37% HCl to pH = 1.0, and the separated oil was extracted with EtOAc (3 × 7 mL). The combined EtOAc extracts were washed with water (3.0 mL), brine (2 × 5 mL) and then dried (MgSO_4_). The solvent was removed in vacuo and the residue was dried in vacuo at 1.0 Torr at 40 °C for 1 h. The obtained foam was dissolved in glacial AcOH (3 mL), then anisole (0.2 mL) and 35% HBr/AcOH (2.2 mL) were added. The reaction mixture was incubated at 20 °C for 1.5 h (until the end of the evolution of CO_2_), pooled into abs. Et_2_O (60 mL) and left overnight at −20 °C. The solvents were decantated, the residual oil was co-evaporated in vacuo with water (2 × 10 mL), the residue was dissolved in water (10 mL) and then applied on a Dowex 50W-X8 column (V = 12 mL). A column elution was performed with water (600 mL), collecting 10 mL fractions, and then ninhydrin-positive fractions (from 15 to 50) were combined, evaporated to dryness in vacuo, and the residue was then dried in vacuo over P_2_O_5_ to give *L*-Leu-*D*,*L*-Glu-γ-P_H_ (640 mg, yield 76% for two steps) as a colorless solid, with an *R_f_* 0.66 on TLC. ^1^H NMR (300.13 MHz, D_2_O): δ = 7.02 (dm, 1H, ^1^*J*_HP_ 514.1 Hz, H-P), 4.57–4.47 (m, 1H, CH-COOH), 4.17–4.09 (m, 1H, CH-NH_2_), 2.24–2.12 (m, 1H, CH_a_-P), 2.09–1.95 (m, 1H, CH_b_-P), 1.90–1.62 (m, 5H, CH_2_-CH_2_-P, CH_2_-CH-NH_2_, CH-(CH_3_)_2_), and 1.10–1.00 (m, 6H, CH-(CH_3_)_2_). ^13^C NMR (75.43 MHz, D_2_O): δ = 177.81 and 177.31 (2 × s, COOH), 173.31 and 173.20 (2 × s, CONH), 56.52 and 56.26 (2 × d, ^3^*J*_PC_ 16.5 Hz and ^3^*J*_PC_ 16.4 Hz, CH-COOH), 54.79 and 54.61 (2 × s, CH-NH_2_), 42.59 (s, CH_2_-CH-NH_2_), 30.25 and 30.05 (2 × d, ^1^*J*_PC_ 89.3 Hz, ^1^*J*_PC_ 89.4 Hz, CH_2_-P), 26.71 and 26.50 (2 × s, CH_2_-CH_2_-P), 25.82 and 25.79 (2 × s, CH-(CH_3_)_2_), 24.39 and 24.35 and 23.94 and 23.91 (4 × s, CH_3_).^31^P NMR (121.44 MHz, D_2_O): δ = 29.34 and 29.17 (2 × s). The symbol “×” indicates differences of the same signals and coupling constants of *L*,*L*- and *L*,*D*-diastereomers. HRMS (ESI-MS): found *m*/*z* 281.1261; calc. for C_10_H_21_N_2_O_5_P [M+H]^+^ 281.1266.

### 2.3. Synthesis of L-Leucyl-D,L-Glu-γ-P_5_

This dipeptide was prepared as described for *L*-*L*eu*-D*,*L*-Glu-γ-P_H_ (see Section 2.2) starting from D,L-AP4 (366 mg, 2.0 mmol) and *N*-Cbz-*L*-Leu-OSu (716 mg, 2.0 mmol) in a H_2_O-1,2-dimethoxyethane mixture. After the deprotection of the crude N-Cbz-dipeptide with 35% HBr/AcOH and the removal of the access of HBr/AcOH as described in Section 2.2, the residue was dissolved in H_2_O (10 mL) and applied on a Dowex 50WX8 column (V = 12 mL). The column was eluted with water (700 mL), collecting 10 mL fractions, and then ninhydrin-positive fractions (from 25 to 60) were combined, evaporated to dryness in vacuo and the residue was then dried in vacuo over P_2_O_5_ to afford *L*-Leucyl-*D*,*L*-Glu-γ-P_5_ (320 mg, with a yield of 54% for two steps): R_f_ 0.29. ^1^H NMR (300.13 MHz, D_2_O): δ = 4.44–4.32 (m, 1H, CH-COOH), 4.00 (dd, 1H, ^3^J_HHa_ 7.5 & 7.4 Hz, ^3^J_HHb_ 7.4 & 6.7 Hz, CH-NH_2_), 2.15–2.01 (m, 1H, CH_a_-P), 2.00–1.85 (m, 1H, CH_b_-P), 1.77–1.54 (m, 5H, CH_2_-CH_2_-P, CH_2_-CH-NH_2_, CH-(CH_3_)_2_), and 0.98–0.85 (m, 6H, CH-(CH_3_)_2_). ^13^C NMR (75.43 MHz, D_2_O): δ = 178.12 and 177.55 (2 × s, COOH), 173.30 and 173.19 (2 × s, CONH), 56.84 and 56.50 (2 × d, ^3^J_PC_ 17.5 Hz and ^3^J_PC_ 17.3 Hz, CH-COOH), 54.81 and 54.62 (2 × s, CH-NH_2_), 42.58 (s, CH_2_-CH-NH_2_), 27.90 and 27.88 (2 × s, CH-(CH_3_)_2_), 26.94 and 26.68 (2 × d, ^1^J_PC_ 134.5 Hz and ^1^J_PC_ 134.7 Hz, CH_2_-P), 26.70 and 26.50 (2 × s, CH_2_-CH_2_-P), 24.39 and 24.35 and 23.93 and 23.91 (4 × s, CH_3_). ^31^P NMR (121.44 MHz, D_2_O): δ = 24.93. HRMS (ESI-MS): found *m*/*z* 297.1210; calc. for C_10_H_21_N_2_O_6_P [M+H]^+^ 297.1215.

### 2.4. The Microdilution Method to Determine the Antimicrobial Activity of Tested Compounds against Escherichia coli

The minimum inhibitory concentration able to inhibit 90% (MIC_90_) of the the growth of the bacterial population of the test strain *E. coli* K12 MG1655 was calculated using the broth microdilution method in the minimal medium EG containing MgSO_4_•7H_2_O (0.2 g), citric acid•H_2_O (2.0 g), anhydrous K_2_HPO_4_ (10.0 g), NaNH_4_HPO_4_•H_2_O (3.5 g), and glucose (4.0 g), milliQ water (1.0 L), at a final pH of 7.0 as described elsewhere [19]. Briefly, overnight cultures (2 mL) of the *E. coli* K12 strain MG1655 grown in LB (lysigeny broth) medium were centrifuged at 3500 rpm for 15 min at 15 °C and the bacterial cellular pellets resuspended in an isovolume of saline solution (9 g/L NaCl). The OD_600_ was then brought to 1.0. The resuspension of the bacterial cells was used to inoculate (1:25) 2 mL of fresh minimal medium EG and the bacteria were allowed to grow for 6 to 7 h at 37 °C from a starting OD_600_ = 0.04 to a final OD_600_ = 0.5 (corresponding to 2.5 × 10^8^ colony forming units, cfu/mL), then diluted (1:25) to a final OD_600_ = 0.02 (corresponding to 1.0 × 10^7^ cfu/mL) in the same minimal medium. This dilution was the one used to set up the 96-well microplate containing a geometrically increasing concentration of the compounds to be tested. In the microplate, 20 µL of bacterial culture (OD_600_ = 0.02) were added to a final volume of 200 µL. Thus, a 1:10 dilution was made and the starting OD_600_ in the microplate reader was 0.002, corresponding to a number of cfu/mL at time zero, as assessed by plating on LB-agar, between 0.5–1.0 × 10^6^/mL. This corresponds to the optimum starting number of cfu/mL to perform an MIC experiment. The microplate was incubated at 37 °C for 24 h in the microplate reader Varioskan Lux (ThermoFisher Scientific, Monza, Italy). The OD_600_ was recorded automatically every hour. Before each reading, the microplate was set to shake vigorously for 10 s, to ensure an even distribution of the bacteria in solution. The MIC_90_ was calculated at 22 h from the inoculum using the equation: % inhibition = [1 − (OD_600_treated/OD_600_untreated)] × 100.

Unless otherwise specified, all the tested compounds were dissolved in Milli-Q water, pH-adjusted to 7–8 by adding 5.0 N NaOH, filtered, dispensed in aliquots and stored at −20 °C.

### 2.5. The Agar Diffusion Method to Analyze Antimicrobial Activity of L-Leucyl-D,L-Glu-γ-P_H_ against Bacillus subtilis ATCC 6633

Different amounts of *L*-Leu-*D*,*L*-Glu-γ-P_H_ were applied to paper disks. The disks were dried in air and placed on the surface of an agar medium for *B. subtilis* [22], containing Gibco (New York, NY, USA) potato starch (25.0 g), glycerol (2.5 g), *L*-Asp (2.0 g), *D*,*L*-Met (0.4 g), K_2_HPO_4_ (6.0 g), KH_2_PO_4_ (2.0 g), NH_4_Cl (1.0 g), NH_4_NO_3_ (0.2 g), Na_2_SO_4_ (0.2 g), MgSO_4_•7H_2_O (0.04 g), MnSO_4_•4H_2_O (0.002 g), FeSO_4_•7H_2_O (0.002 g), CaCl_2_ (0.001 g), agar (15 g), and milliQ water (1.0 L), with a final pH of 6.8, with a seeded lawn of *B. subtilis* ATCC 6633 strain with a seeding density of 10^6^ bacteria per 1 cm^2^ of agar surface. The dishes were incubated for 20 h at 37 °C. Amoxicillin (2.0 μg per disk) was used as a control. The antibiotic activity was determined by the agar diffusion method based on the presence and size of non-growth zones around the disks [23].

## 3. Results

### 3.1. Synthesis of L-Leu-D,L-Glu-γ-P_H_ and L-Leu-D,L-Glu-γ-P_5_

**Scheme 1:**
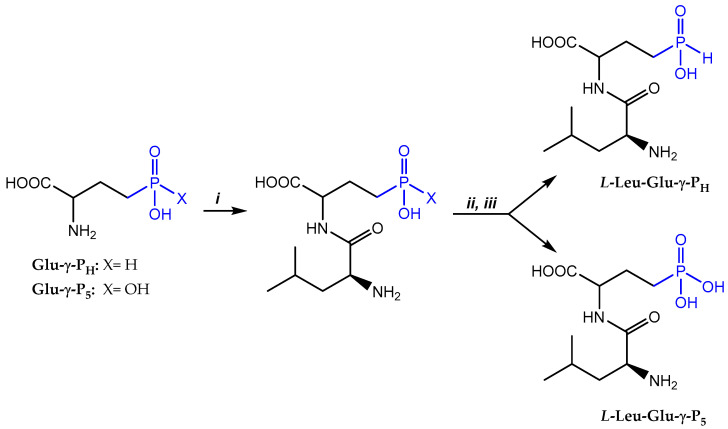
*i*—Cbz-*L*-Leu-OSu/1,2-dimethoxyethane/H_2_O/NaHCO_3_; *ii*—HBr/AcOH; *iii*—Dowex 50 × 8 (H^+^), elution with H_2_O.

The choice for *L*-Leu as a *N*-terminal amino acid was based on the evidence that, once inside the cell, the dipeptide becomes cleaved by peptidases of which *L*-Leu-aminopeptidase is one of the major peptidases responsible for the cleavage. This peptidase has indeed a preference for *L*-Leu in the *N*-terminal position [24]. The synthesis, proceeded by the condensation of *N*-hydroxysuccinimide ester of the *N*-Cbz-*L*-Leu (*N*-Cbz-*L*-Leu-OSu) with Glu-γ-P_H_ or Glu-γ-P_5_ in a water/1,2-dimethoxyethane mixture in the presence of NaHCO_3_/Na_2_CO_3_, was followed by the one-pot removal of the Cbz-protecting group with HBr/AcOH (Scheme 1). Each dipeptide was isolated by ion-exchange chromatography on a sulfocationite Dowex-50W-X8 (H^+^-form), eluting the resin with a large volume of water; however, the water elution did not allow for the separation of the diastereomeric dipeptides, in contrast to what was obtained with *L*-Leu-PT [14]. *L*-Leu-*D*,*L*-Glu-γ-P_H_ was obtained with a 76% overall yield, while the *L*-Leu-*D*,*L*-Glu-γ-P_5_ had a 54% overall yield; thus, compared with the Glu-γ-P_5_, the *H*-phosphinic Glu-γ-P_H_ proved to be more reactive. It should be noted that some hydrogen and carbon atoms of the *L*,*D*- and *L*,*L*-diastereomers of *L*-Leu-*D*,*L*-Glu-γ-P_H_ and *L*-Leu-*D*,*L*-Glu-γ-P_5_ have different chemical shifts in the ^1^H- and ^13^C-NMR spectra (the original spectra are provided in the Supplementary Materials; Figures S1–S4); however, only for the *L*-Leu-*D*,*L*-Glu-γ-P_H_ but not for the *L*-Leu-*D*,*L*-Glu-γ-P_5_ were two characteristic signals corresponding to *L*,*D*- and *L*,*L*-diastereomers detected in ^31^P-NMR spectra (the original spectra are provided in the Supplementary Materials; Figure S5). This finding was unexpected and, to exclude an artifact, we prepared *L*-Ala-*D*-Ala-P_5_ (File S1, Scheme S1), using commercially available *L*-Ala-*L*-Ala-P_5_ and compared the ^1^H- and ^31^P-NMR spectra. In this case, *L*,*D*- and *L*,*L*-diastereomers could be easily distinguished by ^1^H-NMR, but again only one signal was observed in the ^31^P-NMR spectrum (the original spectra are depicted in the Supplementary Materials; Figures S5–S7).

The two newly synthesized *L*-Leu-*D*,L dipeptides, as well as the previously synthesized *L*-Leu-*D*,L-PT [14], were tested for their antibacterial activity on *E. coli*. At this stage of our study we decided not to separate the *L*-L and *L*-D diastereomers because natural and phosphorous-containing dipeptides having one or more *D*-residues are known to be poorly accumulated by *E. coli* due to a stereochemical preference of Dpp for dipeptides containing *L*-residues [25,26].

### 3.2. Continuous Monitoring during MIC Assays in Minimal Medium EG Showed Unusual Growth Behaviour in Stationary Phase

**Figure 2:**
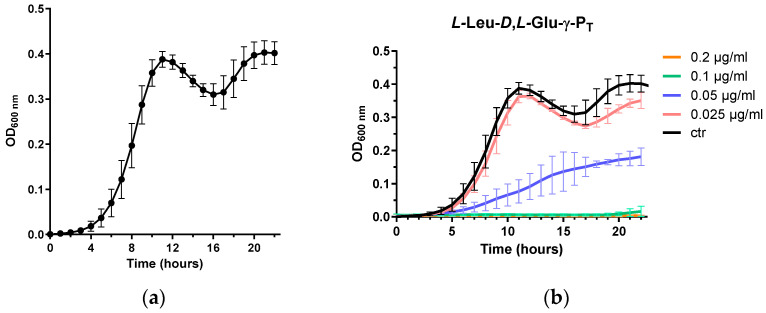
Growth of *E. coli* MG1655 in chemically-defined EG medium. (**a**) Curve obtained by averaging 25 growth curves of the control condition (i.e., MG1655 in the absence of treatment); (**b**) example of the experiments (three biological replicates) with one of the tested dipeptides (see Section 3.3). Data are shown as mean ±SD.

The growth studies presented here were performed using the Varioskan Lux microplate reader (Thermo Scientific, Waltham, MA, USA) that allows simultaneous incubation while monitoring growth. After 24 h, we observed that the *E. coli* strain MG1655, used in our assays, demonstrated a growth profile resembling “diauxic growth” (Figure 2). This phenomenon was missed initially because the growth was only monitored between the 13th and 20th hour, thus missing the initial peak at the 10th hour (indeed we were mostly observing a slight decline of the stationary phase OD_600_). Additionally, previous measurements were taken hourly by manually transferring the microplate from the incubator at 37 °C to the microplate reader (Sunrise Tecan, Männedorf, Switzerland). All these technical differences and the more stable readings performed in the Varioskan Lux microplate reader explain why this growth behaviour, shown in Figure 2, had originally escaped observation.

Basically, following the early lag phase, the strain demonstrated exponential growth and then entered the stationary phase, after which a decrease in the OD_600_ was followed by a restart of the growth to attain an OD_600_ value close to that the first maximum. According to previous reports [27,28], *E. coli* MG1655 might display this behaviour in minimal medium as a consequence of a metabolic switch: basically during the first 10 h, glucose (in this work 0.4%, corresponding to approx. 22 mM) is used as the primary energy source, after which acetate becomes the primary energy source for the bacteria. Acetate in fact is produced and released (approx. 6 mM) in the medium by the bacteria starting from glucose, but is then used as a carbon source at a later stage, i.e., only after glucose depletion [27,28]. This explains the observed re-growth phase, which reached the stationary phase within a few hours, as the acetate concentration available was lower than that of the initial glucose concentrations. Therefore, the MIC_90_ was determined at 22 h as a more representative indicator of the effects of the tested molecules on the growth of *E. coli* MG1655, as illustrated in Figure 2b.

### 3.3. L-Leu-Glu-γ-P_H_ Is a More Potent Inhibitor of the Growth of E. coli K12 Than L-Leu-Glu-γ-P_5_

It is known that aminophosphonic acids are poorly accumulated by bacteria [9], and the *D*,*L*-Glu-γ-P_5_ was not an exception, i.e., it did not inhibit the growth of *E. coli* in a chemically-defined minimal medium when tested at in a range of concentrations up to 2000 µg/mL (Table 1). By contrast, the *D*,*L*-Glu-γ-P_H_ displayed excellent antimicrobial activity (bacteriostatic) against *E. coli* under the same assay conditions (MIC_90_ 20 µg/mL; Table 1; [19]). As reported previously, the *L*-isomer was almost twice as active as the racemic compound (Table 1; [19]), whereas the *D*-isomer was totally inactive under the conditions used in our assays.

Notably, the dipeptide *L*-Leu-*D*,*L*-Glu-γ-P_H_ was found to be 10 times more active than *D*,*L*-Glu-γ-P_H_ against *E. coli* in the same medium, displaying a MIC_90_ 2.0 µg/mL (Table 1). These differences in the activities of *D*,L-Glu-γ-P_H_ and *L*-Leu-*D*,*L*-Glu-γ-P_H_ can be most likely explained by the ability of the dipeptide to more easily penetrate into the cell via the dipeptide permease Dpp. Then, the cytoplasmic cleavage of *L*-Leu-*D*,*L*-Glu-γ-P_H_ likely releases *L*-Glu-γ-P_H_, which can either be a substrate for some enzymes of glutamate metabolism, thereby producing a number of new biologically active phosphorus-containing compounds, or act as an inhibitor in some enzymatic transformations involving glutamate as a substrate (see Discussion).

When the *D*,*L*-Glu-γ-P_5_ was tested, it did not display any antibacterial activity in our assay conditions, whereas its dipeptide derivative, namely, *L*-Leu-*D*,*L*-Glu-γ-P_5_, exhibited antibacterial activity in minimal medium (MIC_90_ 80 µg/mL, Table 1). This clearly indicated that in the case of the phosphonic compound, its incorporation in a dipeptide with *L*-Leu improved its penetration in the microbial cell and its growth inhibitory effects as compared to the free amino acid form.

It should be noted that both the *L*-Leu-*D*,*L*-Glu-γ-P_H_ and *L*-Leu-*D*,*L*-Glu-γ-P_5_ were less effective when tested in a liquid-rich medium (Mueller-Hinton broth). In this medium, the MIC_90_ values for both dipeptides were >>2000 μg/mL, which is in agreement with the literature demonstrating poor antibacterial activity of phosphonopeptides in a nutrient rich medium, likely due to the large content of dipeptides and oligopeptides present in the growth medium [9]. Our results also demonstrated that at high concentrations, the dipeptides’ inhibitory activity was still measurable, whereas that of the active compounds in the free amino acid form was not. This again points to an improved efficacy of the tested compounds when used as dipeptides.

Strikingly, the PT containing dipeptide, the detailed synthesis of which was recently published [14], was >1000 times more effective than racemate PT. This is in line with our previous study where we demonstrated that the *L*-isomer of PT was more potent on *E. coli* and multidrug resistant strains of *K. pneumoniae* when incorporated in a dipeptide with *L*-Leu [14].

### 3.4. L-Leu-D,L-Glu-γ-P_H_ Effectively Inhibits the Growth of Bacillus subtilis ATCC 6633

**Figure 3:**
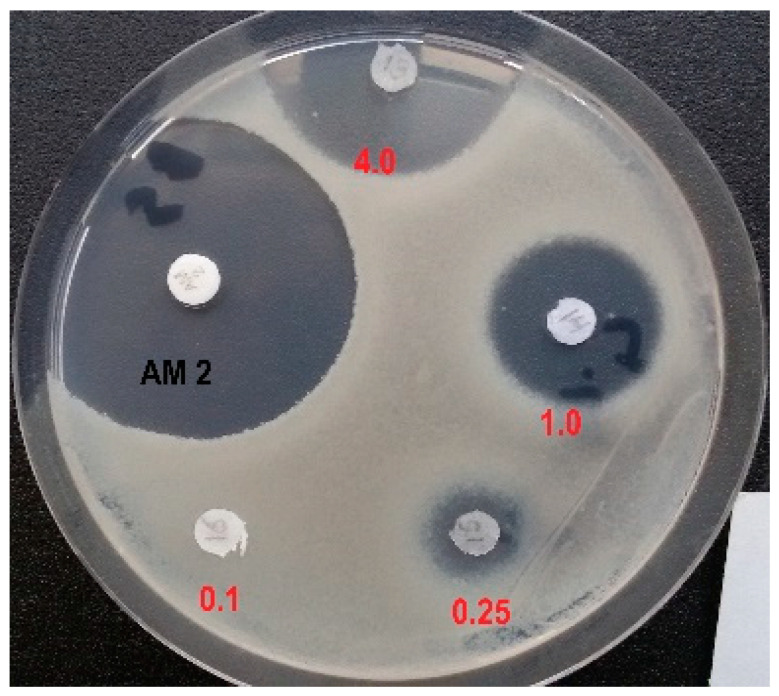
Inhibition of the growth of *B. subtilis* ATCC 6633 with *L*-Leu-*D*,*L*-Glu-γ-P_H_ (tested from 4.0 μg to 0.1 μg/disk) and Amoxicillin (2.0 μg/disk).

Next, we examined the effects of *D*,*L*-Glu-γ-P_H_ and *L*-Leu-*D*,*L*-Glu-γ-P_H_ on the growth of *B. subtilis* ATCC 6633 using the agar-diffusion test. The *D*,*L*-Glu-γ-P_H_ was not active on *B. subtilis* at 100 µg/disk. In striking contrast, the dipeptide *L*-Leu-*D*,*L*-Glu-γ-P_H_ inhibited the growth of *B. subtilis* ATCC 6633 in a dose-dependent manner, starting from 0.25 µg/disk (Figure 3). These differences are likely the result of different transport efficiencies between the *D*,*L*-Glu-γ-P_H_ and *L*-Leu-*D*,*L*-Glu-γ-P_H_ in bacteria. As observed in *E. coli*, the dipeptide actively penetrated *B. subtilis* ATCC 6633 using the dipeptide permease Dpp and released *L*-Glu-γ-P_H_ upon cleavage in the cytoplasm. When compared with Amoxicillin (2.0 μg/disk, Figure 3), a β-lactam antibiotic that interferes with the synthesis of the peptidoglycan, the antibacterial activity of the *L*-Leu-*D*,*L*-Glu-γ-P_H_ was only slightly lower (4 µg/disk; Figure 3); however, it is important to note here that the cytoplasmic cleavage of *L*-Leu-*D*,*L*-Glu-γ-P_H_ released both enantiomers in the cytoplasm, but only the *L*-enantiomer was reported to possess antimicrobial activity [19]. Therefore, the amount of active diastereomer *L*-Leu-*L*-Glu-γ-P_H_ was calculated to be 2.0 μg/disk, which confirmed that the dipeptide *L*-Leu-*L*-Glu-γ-P_H_ exhibited an antimicrobial activity comparable to that of Amoxicillin.

## 4. Discussion

The substitution of the carboxyl group of amino acids with a phosphorus-containing group leads to two main families of analogues, namely, the phosphinic and the phosphonic organophosphorous compounds. The latter -P(O)(OH)_2_ group has a tetrahedral spatial organization, with a double negative charge at a neutral pH, and is unable to mimic the planar single-charged carboxyl group of the amino acids, as we already demonstrated by modelling studies [19]. In agreement, aminophosphonic acids are reported to be poor inhibitors of the enzymes of amino acid metabolism [29]; however, among the derivatives of aminophosphonic acids, i.e., esters, amides and the compounds with a C-P-C backbone, which are stable mimics of tetrahedral intermediates (or reaction transition states) of carboxyl group transformations, there are not only potent enzyme inhibitors, but even commercial drugs [9,29]. Notable examples are the compounds listed in the Introduction (with some depicted in Figure 1a) including PT, which inhibits GS [10,11]. Another notable example is a peptidomimetic containing a phosphonate moiety in place of the peptide bond, namely, Fosinopril^®^, which acts as an inhibitor of the zinc-dependent angiotensin-converting enzyme and is used to treat hypertension [30].

By contrast, the substitution of one hydroxyl group of aminophosphonic acids with a hydrogen atom eliminates one negative charge as well a bulky atom (i.e., oxygen) and confers to the *H*-phosphinic group a flattened tetrahedral geometry as suggested by crystallographic data for the β-*H*-phosphinic analogue of aspartate [31]. The *H*-phosphinic group can indeed be considered as a bioisostere of the carboxyl group, as confirmed by several substrate-like transformations of α-amino-*H*-phosphinic acids ([19] and ref. therein).

**Figure 4:**
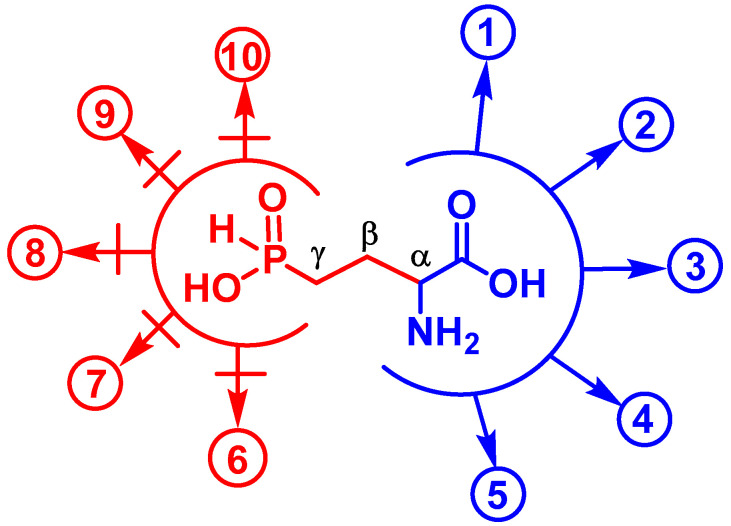
Some enzymatic transformations of *L*-Glu which are potentially affected by *L*-Glu-γ-P_H_. The two pharmacophores (C-α and phosphinic group) are delimited by an open circle. Substrate-like transformations of *L*-Glu-γ-P_H_ (in blue): (1) formation of 2-oxo-4-phosphinobutyric acid (α-KG-γ-P_H_) via PLP-dependent transamination (*L*-Glu-γ-P_H_ as an amino group donor); (2) formation of α-KG-γ-P_H_ in glutamate dehydrogenase reaction (oxidative deamination); (3) H^+^-dependent decarboxylation of *L*-Glu-γ-P_H_, yielding a *H*-phosphinic analogue of GABA; (4) formation of *L*-Glu-γ-P_H_-tRNA^Glu^ in a glutamyl-tRNA synthetase reaction; (5) PLP-dependent racemization of *L*-Glu-γ-P_H_ yielding *D*-Glu-γ-P_H_. Reactions in which *L*-Glu-γ-P_H_ cannot be a substrate, but may be an inhibitor (in red): (6) biosynthesis of glutamine; (7) proline; (8) ornithine; (9) glutathione; and (10) dihydrofolate. Reactions (1) and (2) can be reversible. Transformations (6)–(10) include the step of ATP-dependent ligation of Glu and the subsequent transformations of the glutamyl phosphate into the product(s) that are biochemically impossible for *L*-Glu-γ-P_H_.

The demethylphosphinothricin, Glu-γ-P_H_ (Figure 1b), was first discovered and isolated from *Streptomyces hygroscopicus* and *S. viridochromogenes* [15] as a key intermediate of the biosynthetic pathway leading to Bialaphos (a tripeptide containing phosphinothricin, i.e., PT, and two alanyl residues), a well-known commercial herbicide [32]. The key biochemical feature of Glu-γ-P_H_ is the presence of two pharmacophores in the molecule, i.e., the *H*-phosphinic group replacing the γ-carboxyl group and the α-amino acid moiety (Figure 4). This explains the ability of Glu-γ-P_H_ to undergo some substrate-like transformations via α-amino acid moiety leading to metabolites containing unusual C-P-H bonds (see the blue pathways in Figure 4), as recently demonstrated [19]. Some of the de novo synthesized metabolites may be those responsible for the observed antibacterial activity.

It is known that *L*-Glu-γ-P_H_ is a substrate of the PLP-dependent enzyme aspartate aminotransferase, giving rise to a *H*-phosphinic analogue of α-ketoglutarate, i.e., 2-oxo-4-phosphinobutyric acid (hereafter referred to as α-KG-γ-P_H_; Figure 4, reaction 1) [33]. *L*-Glu-γ-P_H_ is a substrate of *E. coli* GABA transaminase producing α-KG-γ-P_H_ [34]. According to our preliminary observations (unpublished), α-KG-γ-P_H_ is also formed in the NAD-dependent glutamate dehydrogenase reaction (Figure 4, reaction 2). As a consequence of one of the above reactions, α-KG-γ-P_H_ may enter the TCA cycle and cause antibacterial activity by acting as an inhibitor of central metabolism. *L*-Glu-γ-P_H_ was also found to be the substrate of the PLP-dependent enzyme glutamate decarboxylase from *E. coli*, yielding the *H*-phosphinic analogue of GABA, GABA-P_H_ (Figure 4, reaction 3), which then undergoes transamination by GABA-transaminase, and the resulting 3-phosphinopropionic aldehyde (the *H*-phosphinic analogue of succinic semi-aldehyde) is then oxidized in a NAD-dependent reaction to the *H*-phosphinic analogue of succinate by succinic semialdehyde dehydrogenase [19]. Since the *H*-phosphinic group is a bioisostere of the carboxyl group, it cannot be excluded that *L*-Glu-γ-P_H_ may be a substrate of glutamyl-tRNA synthetase (Figure 4, reaction 4) with a subsequent formation of peptides carrying a few *L*-Glu-γ-P_H_ residues (though the incorporation of *L*-Glu-γ-P_H_ is not expected to occur to a significant extent because of the competition with the much more abundant *L*-glutamate in the glutamyl-tRNA synthetase reaction). It is also plausible that *L*-Glu-γ-P_H_ undergoes PLP-dependent racemization to generate *D*-Glu-γ-P_H_ (Figure 4, reaction 5) which, however, will not be involved in the biosynthesis of peptidoglycan (murein), an important component of the bacterial cell wall, due to the different chemistry of the carboxyl and *H*-phosphinic groups.

When considering the antibacterial activity of *L*-Glu-γ-P_H_, the contribution of the second pharmacophore, the distal *H*-phosphinic group, must be taken into consideration. Transformations of γ-carboxyl group of the natural amino acid *L*-glutamate lead to the formation of glutamine (for nitrogen assimilation), glutathione (an essential antioxidant), and dihydrofolate (essential in reactions involving the transfer of one-carbon units). Moreover, transformations of the γ-carboxyl group of *L*-glutamate are involved in the biosynthesis of proline and ornithine. All the listed reactions lead to the formation of γ-glutamyl phosphate, or γ-glutamyl adenylate, an intermediate through ATP-dependent ligation, which is a key step in the biosynthesis of the above important metabolites. The intermediate formation of such activated *L*-Glu-γ-P_H_ derivatives is in principle possible, since the *H*-phosphinic analogues of methionine and valine are substrates of the ATP-PPi exchange reaction catalyzed by Met- and Val-aminoacyl-tRNA synthetases [35]; however, the transfer of Met- and Val *H*-phosphinic analogues to the 3′-end of tRNA was not observed and is biochemically impossible because the corresponding enzymes are highly complementary only to the tetrahedral transition state of a carboxyl group, while the transition state of a *H*-phosphinic group is a trigonal bipyramid. These considerations a priori restrict the substrate-like transformations of *L*-Glu-γ-P_H_ via the *H*-phosphinic group (Figure 4, pathways 6–10). Thus, while *L*-Glu-γ-P_H_ may be expected to inhibit the glutamine, glutathione, dihydrofolate, proline, and ornithine biosynthetic pathways, it is difficult to predict how efficient this inhibition would be.

Based on the above, the antibacterial activity of *L*-Leu-*D*,*L*-Glu-γ-P_H_, which penetrates in bacteria via the peptidyl permease system and upon cleavage via peptidases, releases the antibacterial *L*-Glu-γ-P_H_. This may occur either via the biochemical transformation of its α-amino acid moiety (Figure 4), giving rise to new biologically active metabolites containing a C-P-H bond, or by interfering with the transformations at its γ-position due to the presence of a *H*-phosphinic group.

In this work we observed that the phosphonic dipeptide *L*-Leu-*D*,*L*-Glu-γ-P_5_ was significantly less active against *E. coli* when compared to *L*-Leu-*D*,*L*-Glu-γ-P_H_ (Table 1). It is possible that such a difference may be due to their differences in bioavailability, although phosphonopeptides are known to effectively penetrate bacteria using peptidyl permeases [9,26,34]. In our opinion these differences are more likely due to the inability of Glu-γ-P_5_, released in the cytosol after dipeptide cleavage, to undergo substrate-like transformations (reactions 1–5 in Figure 4) that lead to new biologically active phosphonic compounds, which is likely to occur with *L*-Glu-γ-P_H_ (Figure 4). This is in line with the inability of *D*,*L*-Glu-γ-P_5_ to act as either a substrate or an inhibitor of *E. coli* glutamate decarboxylase [19,36] and porcine heart aspartate aminotransferase [33]. On the other hand, the phosphonic group is a doubly-charged tetrahedral group which mimics the tetrahedral intermediates (or reaction transition states) of the carboxyl group [9,29] and this explains the rather high competitive inhibition (K_I_ 50 µM) of *E. coli* glutamine synthetase (GS) with Glu-γ-P_5_ [37]. However, PT (see Introduction and Figure 1a), a naturally occurring inhibitor of GS, can undergo ATP-dependent ligation, with the formed pyrophosphonate that mimics the phosphorylated intermediate of glutamate occurring in the GS-catalyzed reaction [10,11]. Notably, PT has K_I_ 0.6 µM against the *E. coli* GS [38]. These differences in the inhibitory activities of Glu-γ-P_5_ and PT may partly explain the differences observed in the activities of *L*-Leu-*D*,*L*-Glu-γ-P_5_ and *L*-Leu-*D*,*L*-PT against *E. coli*: the first dipeptide has a MIC_90_ = 80 µg/mL, while the second has a MIC_90_ = 0.1 µg/mL (Table 1).

## 5. Conclusions

Antibiotics are chemical substances used to treat bacterial infections in human and veterinary diseases. It is generally agreed that antibiotics’ discovery in the 20th century has revolutionized modern medicine by enabling the treatment of life-threating infectious diseases and allowing major advances in modern medicine, such as surgery and chemotherapy. In the last decades, however, the over- and mis-use of antibiotics, along with the lack of development and innovation in this field has exacerbated the phenomenon of the emergence and spreading of antibiotic resistant bacteria. To avoid a return to the pre-antibiotic era, we urgently need additional antibiotics with new pharmacophores and mechanisms of action.

Given the abundance and the many key roles played by glutamate in microbial metabolism, Glu-γ-P_H_ may affect one or more of the metabolic pathways in which glutamate is involved. For this multi-target potential, which would significantly delay the appearance of resistance mechanisms, Glu-γ-P_H_ is a new promising antibacterial. Notably, acute toxicity studies conducted with Glu-γ-P_H_ on rats and mice have showed very low toxicity [18]. To the best of our knowledge, amino acid derivatives containing a γ-phosphinic group have never been tested as an antibacterial. Our findings, in addition to previously published results, strongly suggest that the antibacterial activity of *L*-Glu-γ-P_H_ can be attributed to the formation of *H*-phosphinic intermediates through substrate-like transformations involving the α-amino acid moiety. The mechanism of action of *L*-Glu-γ-P_H_ is still unknown and its antibacterial activity cannot be directly attributed to a specific pharmacophore, i.e., the α-carbon moiety and/or the γ-phosphinic moiety (Figure 4); however, the present work provides evidence that *H*-phosphinic compounds are attractive molecules with antibacterial activity on a novel metabolic target, with an important potential in treating multidrug-resistant pathogenic microorganisms.

## 6. Patents

A patent IT 102016000098005 has been granted, which includes *L*-Leu-Glu-γ-P_H_, one of the compounds investigated in detail in this study. https://www.uniroma1.it/en/brevetto/102016000098005 (accessed on 22 September 2023).

## Figures and Tables

**Table 1:** Minimum inhibitory concentration (MIC) of the compounds tested on *E. coli* K12 MG1655 determined by the broth microdilution method in chemically-defined minimal medium (EG).

Compound		MIC_90_ (µg/mL)	MIC_90_ (µM)
**Amino acids**	*D*,*L*-Glu-γ-P_H_	20	119.03
	*D*,*L*-PT	266–304 ^1^	1461–1670
	*D*,*L*-Glu-γ-P_5_	n.d. ^2^	n.d.
**Dipeptides**	*L*-Leu-*D*,*L*-Glu-γ-P_H_	2	7.11
	*L*-Leu-*D*,*L*-PT	0.1	0.34
	*L*-Leu-*D*,*L*-Glu-γ-P_5_	80	269.25

^1^ from [14]; ^2^ n.d.: not detectable when assayed at up to 2000 µg/mL.

## Data Availability

Additional data are available in the Supplementary Materials. If not present there, they can be asked for of the authors upon a reasonable and motivated request.

## Supplementary Materials

The following supporting information can be downloaded at: https://www.mdpi.com/article/10.3390/biom13101451/s1. File S1. Synthesis of L-Alanyl-D-Ala-P5; Scheme S1: i—Cbz-L-Ala-OSu/dioxane/H_2_O/NaHCO_3_; ii—HBr/АсОН; iii—Dowex 50W-X8 (H+), elution with H_2_O; Figure S1. 1H-NMR spectrum of L-Leu-Glu-γ-PH; Figure S2. 13C-NMR spectrum of L-Leu-Glu-γ-PH; Figure S3. 1H-NMR spectrum of L-Leu-Glu-γ-P5; Figure S4. 13C-NMR spectrum of L-Leu-Glu-γ-P5; Figure S5. 31P-NMR spectra of L-Leu-Glu-γ-PH (A); L-Leu-Glu-γ-P5 (B); and a mixture (5:3) of L-Ala-L-Ala-P5 and L-Ala-D-Ala-P5 (C); Figure S6. 1H-NMR spectrum of L-Ala-D-Ala-P5; Figure S7. 1H-NMR spectrum of L-Ala-L-Ala-P5 and L-Ala-D-Ala-P5 mixture (5:3).
