# Supplementary material for: A Desmethylphosphinothricin Dipeptide Derivative Effectively Inhibits Escherichia coli and Bacillus subtilis Growth
Source: Biomolecules. 2023 Sep 26;13(10):1451. doi: 10.3390/biom13101451 (PMC10604730; doi:10.3390/biom13101451)
Supplement: Supplementary file 1 [file biomolecules-13-01451-s001.zip › biomolecules-2607938-supplementary.pdf]

Supplementary materials

A desmethylphosphinothricin dipeptide derivative effectively inhibits *Escherichia coli* and *Bacillus subtilis* growth

Maxim A. Khomutov[1†], Fabio Giovannercole[2†§], Laura Onillon[2#], Marija V. Demiankova[3], Byazilya F. Vasilieva[3], Arthur I. Salikhov[1], Sergey N. Kochetkov[1], Olga V. Efremenkova[3]*, Alex R. Khomutov[1]* Daniela De Biase[2]*.

[1] Engelhardt Institute of Molecular Biology, Russian Academy of Sciences, Vavilov Street 32, 119991 Moscow, Russia; makhomutov@mail.ru (M.A.K.); asalihov93@gmail.com (A.I.S.); kochet@eimb.ru (S.N.K.)

[2] Department of Medico-Surgical Sciences and Biotechnologies, Sapienza University of Rome, Corso della Repubblica 79, I-04100 Latina, Italy; fabio.giovannercole@gmail.com (F.G.); laura.onillon@ifremer.fr (L.O.).

[3] Gause Institute of New Antibiotics, Bol'shaya Pirogovskaya 11, 119021 Moscow, Russia; mary_bunny@mail.ru (M.V.D.), bfvas@yandex.ru (B.F.V.)

***** Correspondence: ovefr@yandex.ru (O.V.E.); alexkhom@eimb.ru (A.R.K.) daniela.debiase@uniroma1.it (D.D.B).

[†] M.A.K. and F.G. contributed equally to this work.

[§] Present address: Département de Biologie, Université de Namur, Rue de Bruxelles 61, 5000, Namur, Belgium.

[#] Present address: IHPE UMR 5244, Université de Montpellier, Place Eugène Bataillon CC 80, F-34095 Montpellier Cedex 5, France.

General

D-Ala-P_5 (cat. # 06657) and *L*-Alanyl-*L*-Ala-P_5 (cat. # 05260) were obtained from Fluka; *N*-(benzyloxycarbonyl)-*L*-alanyl *N*-hydroxysuccinimide ester (*Z*-*L*-Ala-OSu) was prepared according to [1] and was recrystallized from *i*-PrOH before use.

TLC was carried out on plastic sheet Cellulose F_{254} (Merck, Germany) in *i*-PrOH–25% NH_4OH–H_2O = 7:1:2. *L*-Alanyl-*D*-Ala-P_5 was detected on TLC plates following staining with ninhydrin (0.4% in acetone).

Ion-exchange chromatography was carried out on Dowex 50WX8, H+-form, 100-200 mesh (BioRad) using water for elution.

NMR spectra were recorded on a Bruker AM-300 (300.13 MHz for ^1H and 121.44 MHz for ^{31}P) using D_2O as a solvent with sodium 3-trimethyl-1-propanesulfonate (DSS) as internal, or 85% H_3PO_4 as external standards. Chemical shifts are given in parts per million (ppm), the letter "*J*" indicates spin-spin coupling constants which are given in Hertz (Hz).

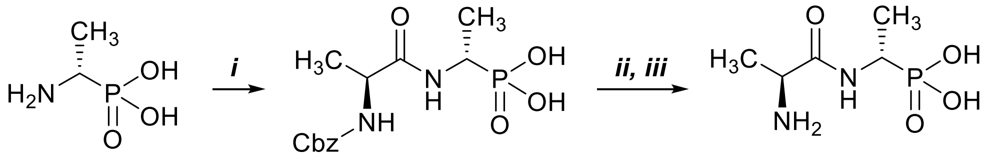

L-Ala-*D*-Ala-P_5

Scheme. ***i***- Cbz-*L*-Ala-OSu/dioxane/H_2O/$NaHCO_3$; ***ii***- HBr/AcOH; ***iii***- Dowex 50X8 (H+), elution with H_2O.

Synthesis of L-Alanyl-D-Ala-P_5

To the solution of *D*-Ala-P_5 (375 mg, 3.0 mmol) in water (10 mL) and 1,4-dioxane (5 mL) containing $NaHCO_3$ (840 mg, 10 mmol), a solution of *N*-Cbz-*L*-Ala-OSu (0.96 g, 3.0 mmol) in 1,4-dioxane (5 mL) was added and the reaction was stirred overnight at 20°C. The reaction mixture was concentrated *in vacuo,* the residue was dissolved in water (15 mL), acidified with 37% HCl to pH 1.0, and the separated oil was extracted with EtOAc (3 x 5 mL). The combined EtOAc extracts were washed with water (3.0 mL), brine (2 x 5 mL) and dried ($MgSO_4$). Solvent was removed *in vacuo* and the residue was dried *in vacuo* at 1.0 Torr at 40°C for 1 h. Thus obtained viscous oil was dissolved in glacial AcOH (1.7 mL); upon the addition of anisole (2 drops) and 35% HBr/AcOH (1.15 mL) the reaction mixture was incubated at 20°C for 1.5 h (until the end of the evolution of CO_2), pooled into abs. Et_2O (30 mL) and left overnight at -20°C. Solvents were decantated, the residual oil was co-evaporated *in vacuo* with water (2 x 10 mL), the residue was dissolved in water (5 mL) and applied on a Dowex 50WX8 column (V= 7.5 mL). Column was eluted with water, collecting 10 mL fractions. Ninhydrin-positive fractions

(from 7 to 16) were pooled and evaporated to dryness *in vacuo*. The residue was recrystallized from water-EtOH and crystals were dried *in vacuo* over P_2O_5 to give *L*-Leu-*D*-Ala-P_5 (165 mg, yield 28% for two steps), R_f 0.22. ^{1}H NMR (300.13 MHz, D_2O): δ = 4.07- 3.88 (m, 2H, >CH-C(O) + >CH-P), 1.48 (d, 3H, $^3J_{HH}$ 7.0 Hz, C<u>H</u>$_3$-CH-C(O)-), 1.25 (dd, 3H, $^3J_{HH}$ 7.3 Hz, $^3J_{HP}$ 14.4 Hz, C<u>H</u>$_3$-CH-P). ^{31}P NMR (121.44 MHz, D_2O): δ = 18.37. For the original spectra see Figure S5 and Figure S6.

References

1. Anderson, G.W.; Zimmerman, J.E.; Callahan, F.M. N-Hydroxysuccinimide Esters in Peptide Synthesis. *Journal of the American Chemical Society* **1963**, *85*, 3039-3039, doi:10.1021/ja00902a047.

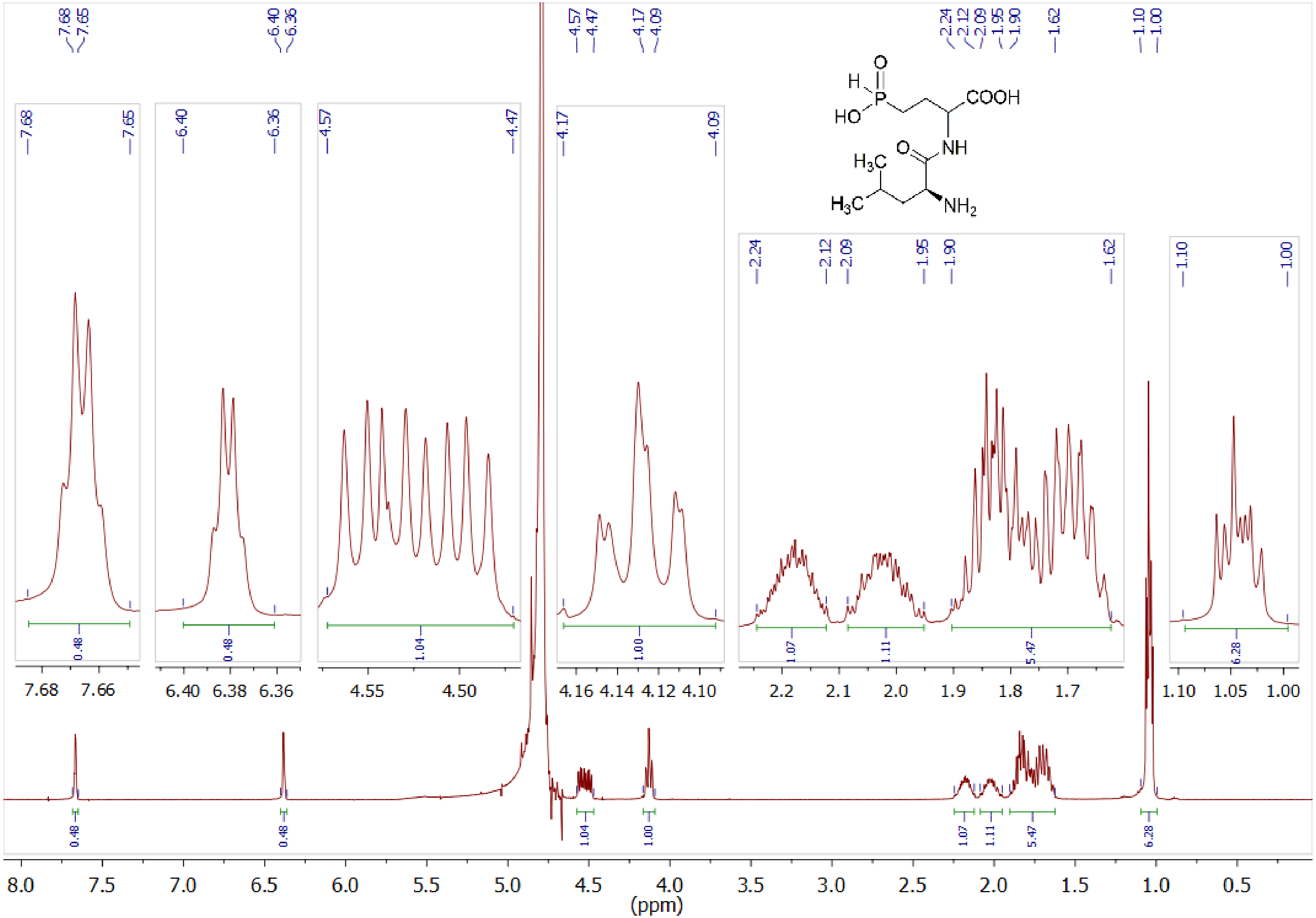

Figure S1. ¹H-NMR spectrum of *L*-Leu-Glu-γ-P_H.

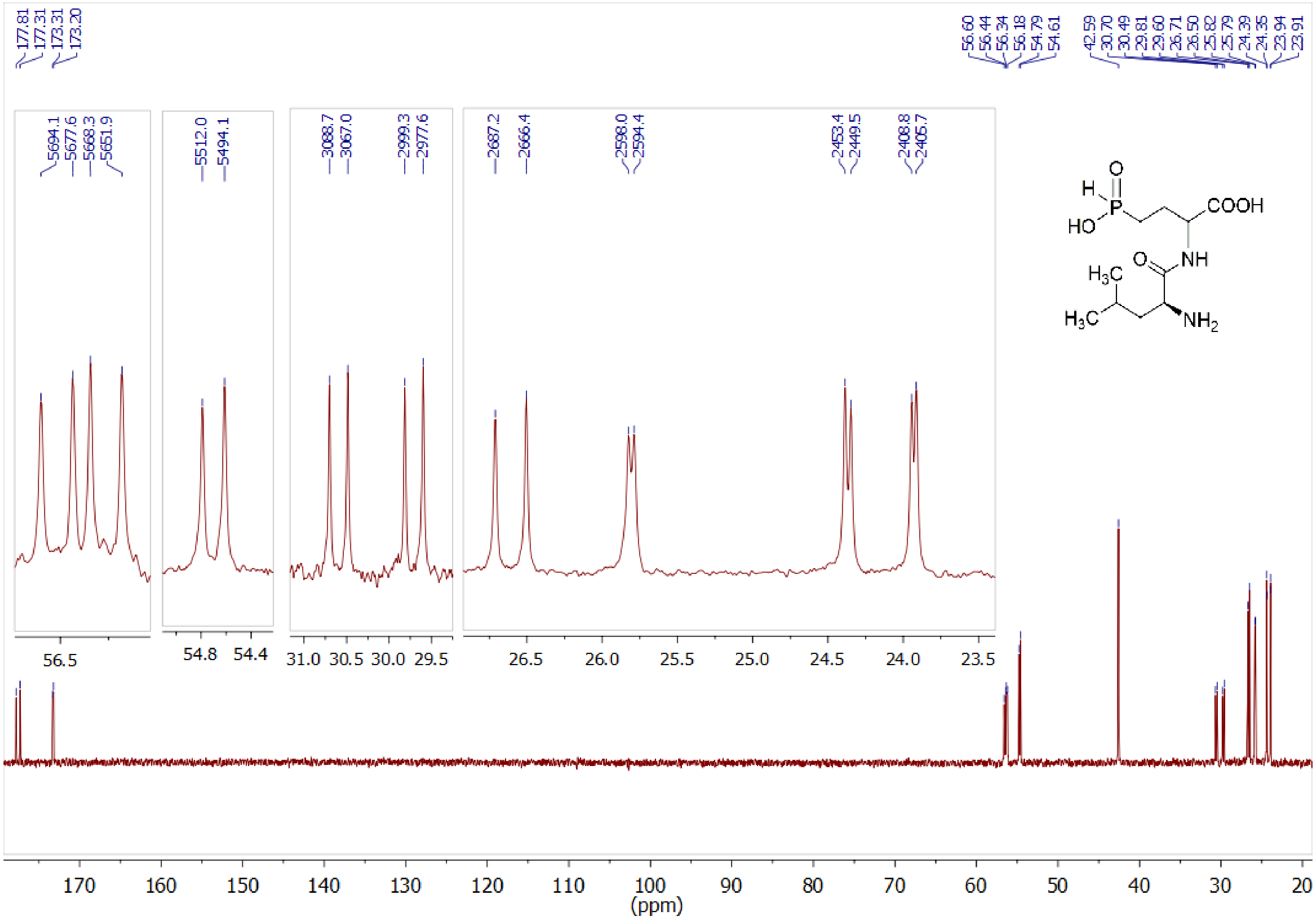

Figure S2. ^{13}C-NMR spectrum of *L*-Leu-Glu-γ-P_H.

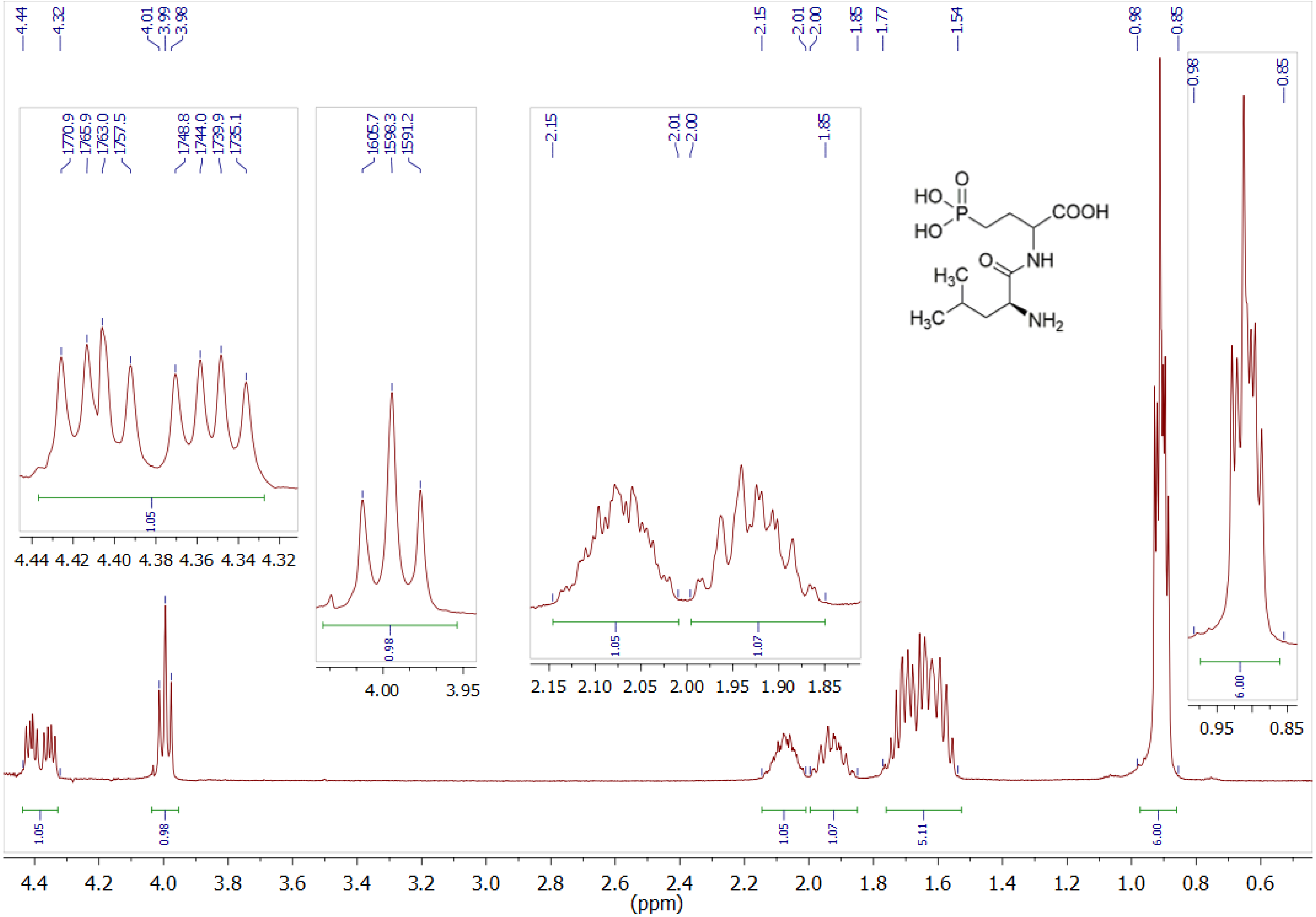

Figure S3. ^{1}H-NMR spectrum of *L*-Leu-Glu-γ-P_5.

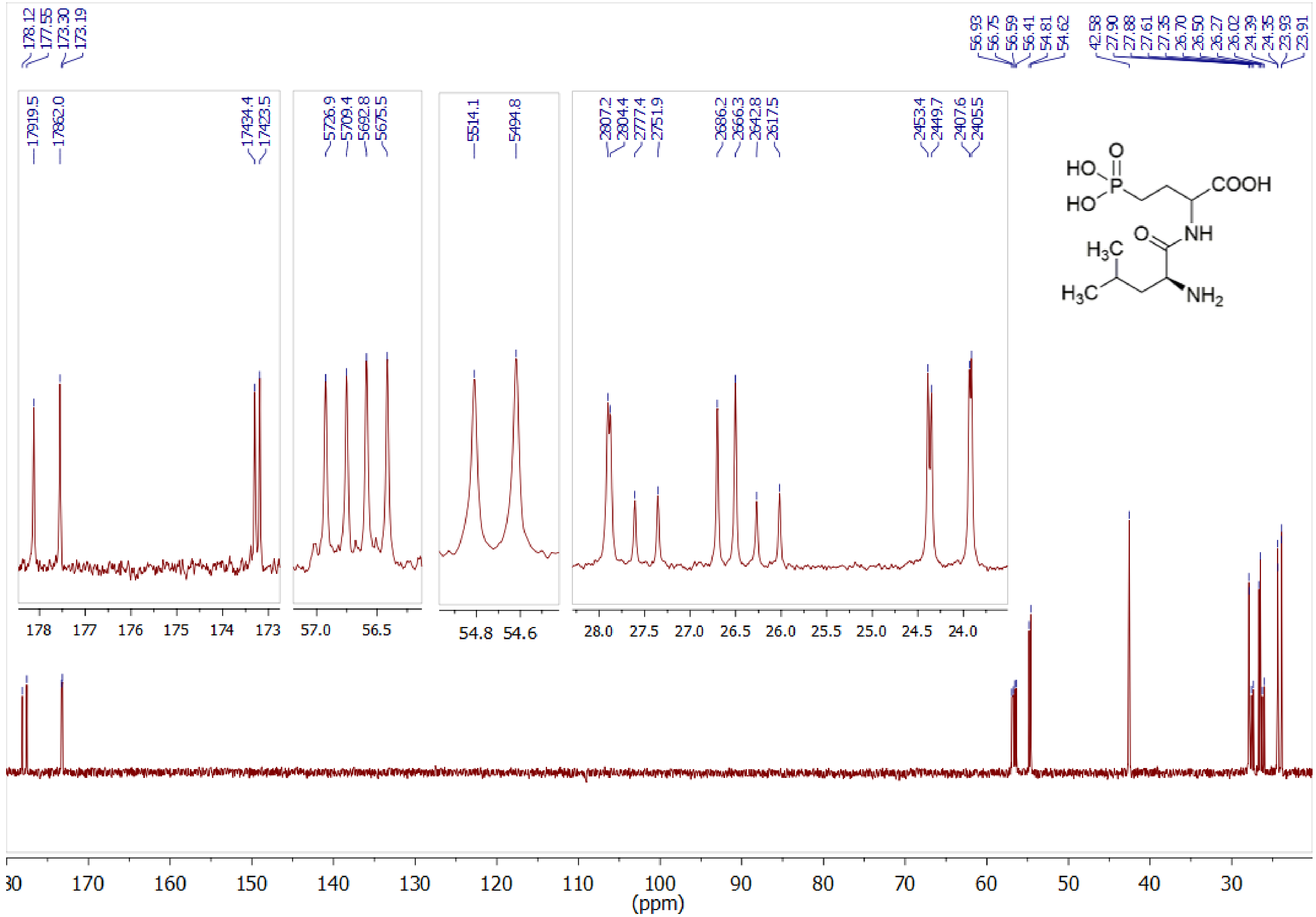

Figure S4. ^{13}C-NMR spectrum of *L*-Leu-Glu-γ-P_5.

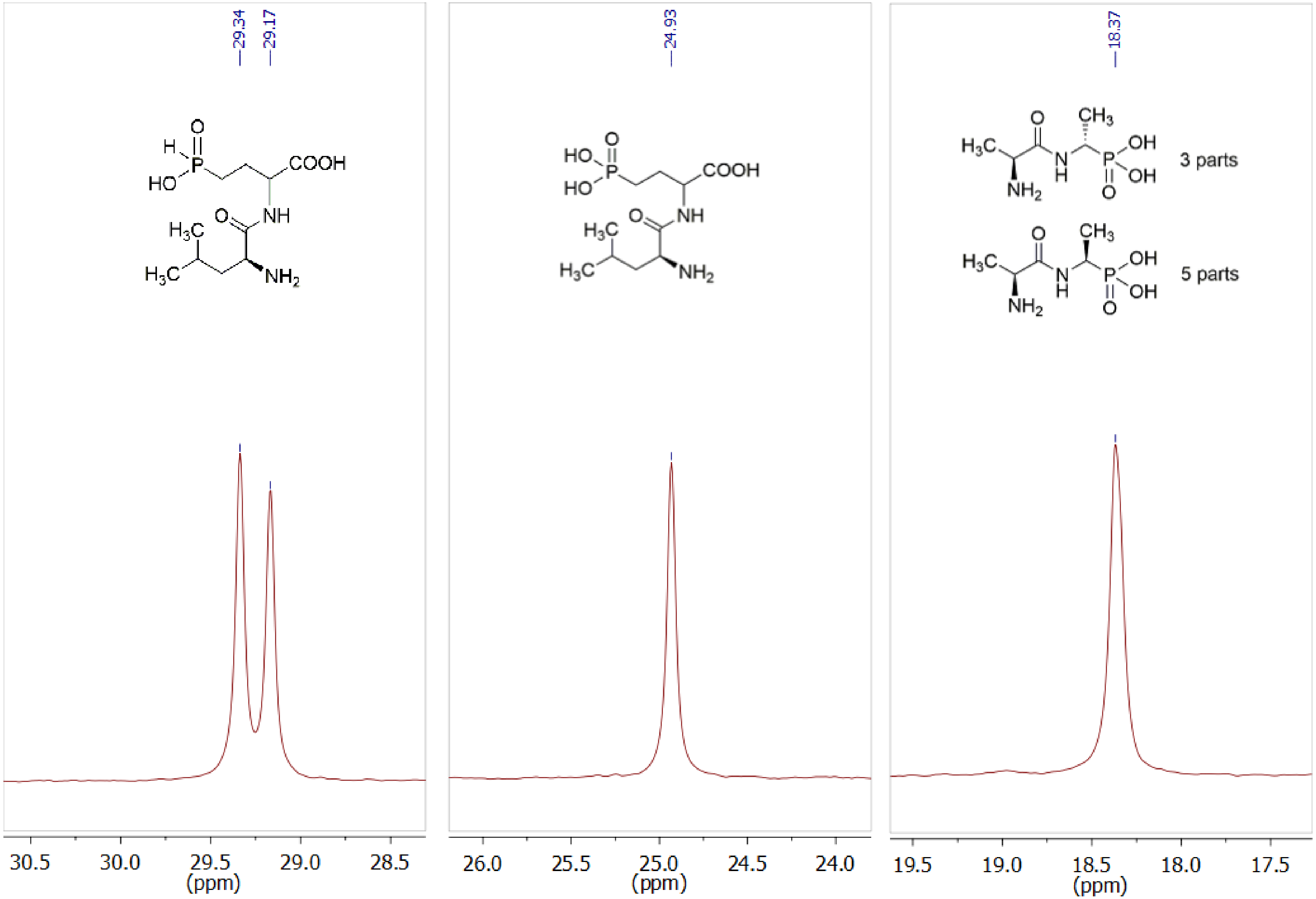

Figure S5. ^{31}P-NMR spectra of *L*-Leu-Glu-γ-P_H (A); *L*-Leu-Glu-γ-P_5 (B); and a mixture (5:3) of *L*-Ala-*L*-Ala-P_5 and *L*-Ala-*D*-Ala-P_5 (C).

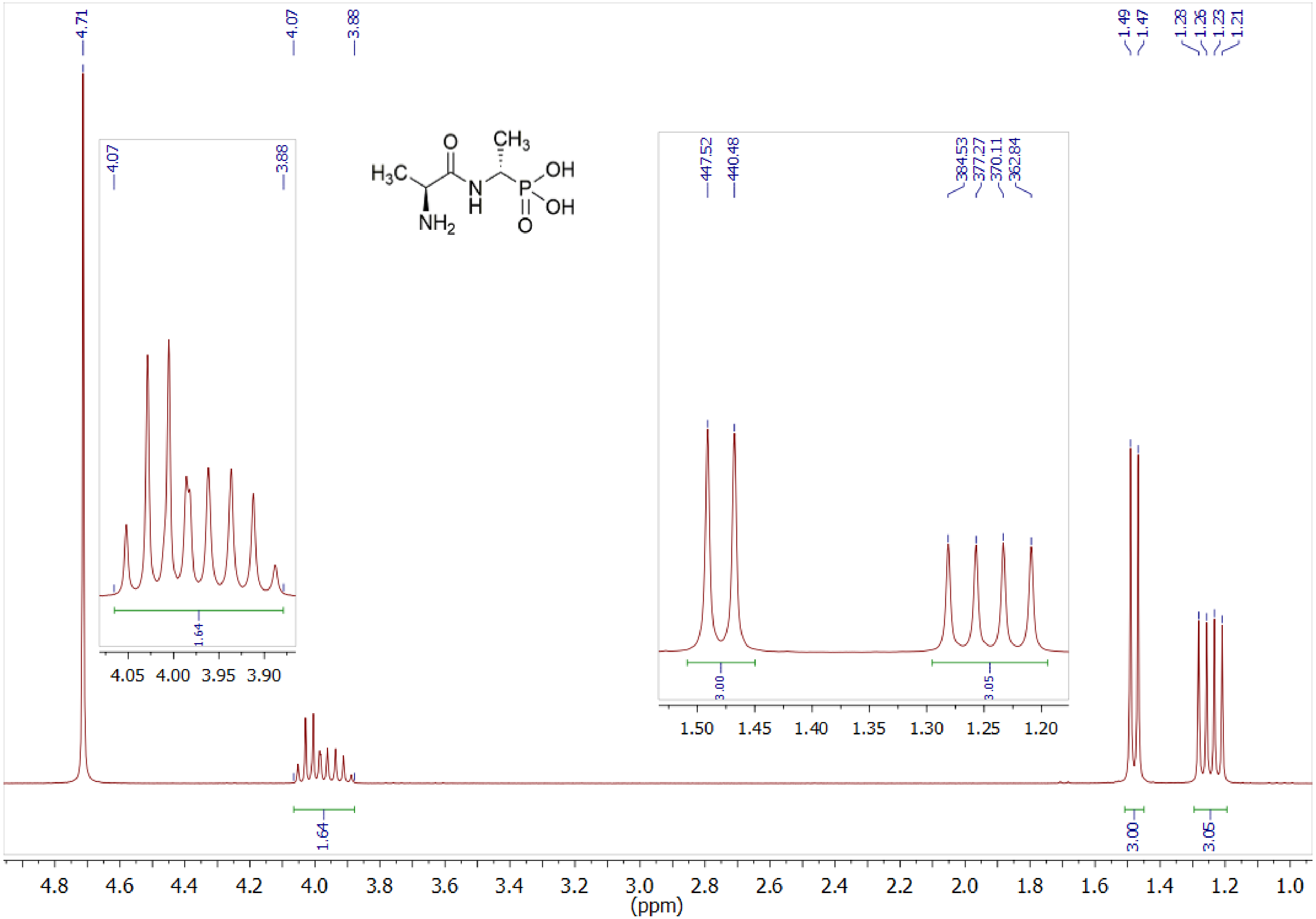

Figure S6. ^{1}H-NMR spectrum of *L*-Ala-*D*-Ala-P_5.

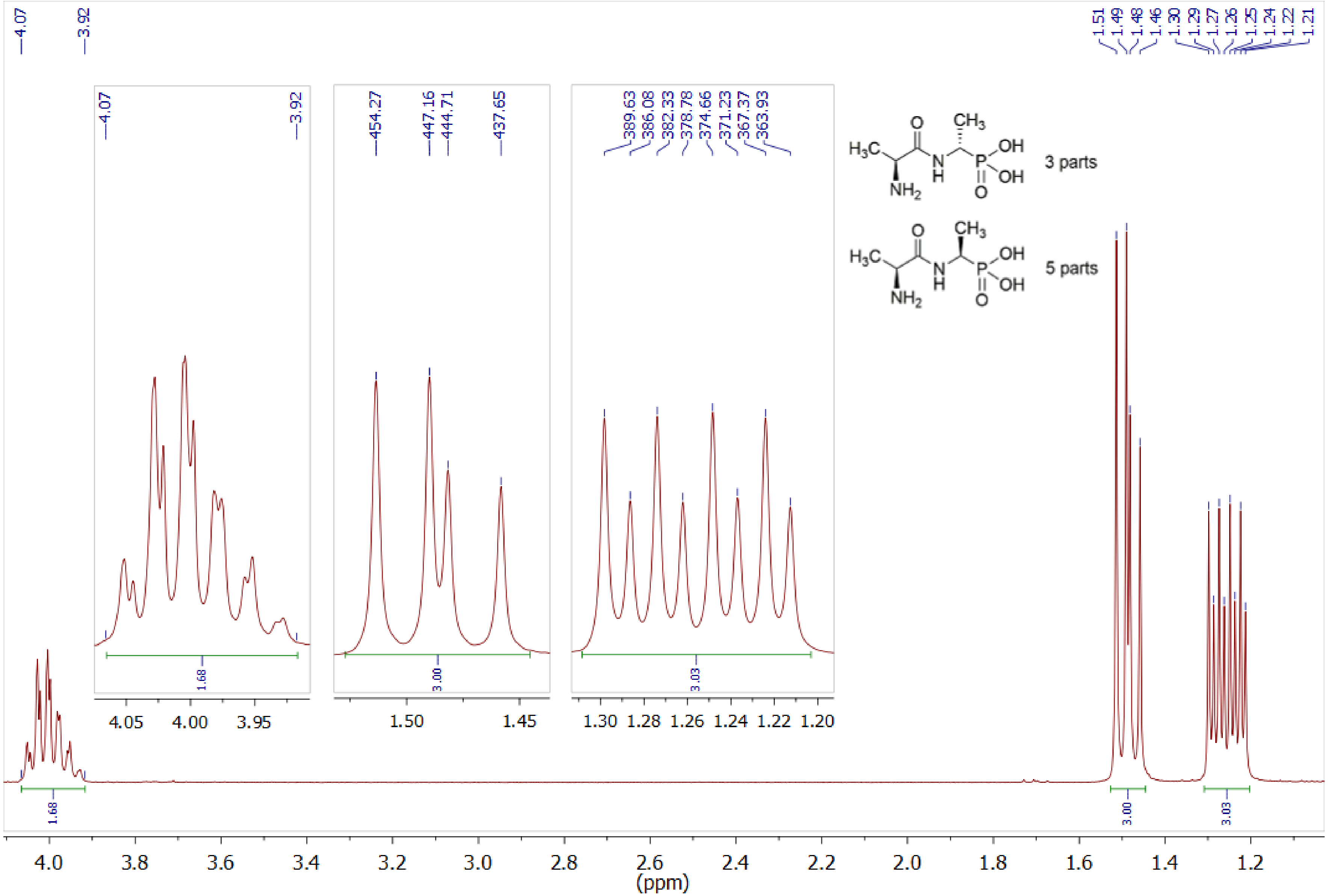

Figure S7. ^{1}H-NMR spectrum of *L*-Ala-*L*-Ala-P_5 and *L*-Ala-*D*-Ala-P_5 mixture (5:3).
